# The “gut microbiota-ferroptosis axis”: a hypothesis for osteoarthritis pathogenesis and therapeutic implications

**DOI:** 10.3389/fmicb.2025.1685962

**Published:** 2025-11-12

**Authors:** Tao Wang, Yuanze Xu, Haorui Zha, Lianguo Wu

**Affiliations:** 1 The Second Clinical Medical College of Zhejiang Chinese Medical University, Hangzhou, Zhejiang, China; 2 The Second Affiliated Hospital of Zhejiang Chinese Medical University, Hangzhou, Zhejiang, China

**Keywords:** osteoarthritis, gut-joint axis, ferroptosis, gut microbiota, therapeutic strategies

## Abstract

Knee osteoarthritis, a common type of osteoarthritis (OA), is a significant driver of global disability. Current treatments offer mainly transient symptomatic relief but fail to halt disease progression. Thus, it is critical to find out useful disease modification methods for OA. Accumulating evidences indicated that iron-dependent regulated cell death called ferroptosis contributes to OA recently. The concept that gut-joint axis including gut microbes and their metabolites may participate in OA progression by linking with local and systemic inflammation might be able to facilitate cartilage degradation. The present review delineates the complex association between ferroptosis and the gut-joint axis in OA. This review synthesizes cross-disciplinary evidence to propose a novel hypothetical framework: the “gut microbiota-ferroptosis axis” as a driver of OA progression. We speculate that abnormal iron metabolism and gut microbiota dysbiosis can promote each other and play a synergistic role in promoting ferroptosis of chondrocytes. When gut microbiota dysbiosis occurs, it can accelerate the onset process of osteoarthritis by consuming protective metabolites that inhibit ferroptosis [such as serotonin (5-HT) and 3-hydroxyanthranilic acid (3-HAA)] and increasing the levels of ferroptosis - promoting compounds [such as lipopolysaccharide (LPS)]. Conversely, reactive oxygen species and lipid peroxides released during ferroptosis can systemically disseminate through blood circulation, exacerbating gut dysbiosis and intestinal barrier dysfunction, thereby establishing a potential self-amplifying loop between ferroptosis and gut dysfunction in OA. Therapeutic interventions targeting this axis, such as resveratrol, and quercetin, show promise by simultaneously modulating gut-joint signaling and suppressing ferroptosis, potentially achieving intestinal barrier recovery and chondrocyte rescue. Future investigations should give priority to dissecting the precise molecular crosstalk within the “gut microbiota-ferroptosis axis” and evaluating combined therapeutic strategies in preclinical models and clinical trials to validate their translational potential for OA.

## 1 Introduction

Globally, knee osteoarthritis (KOA) is one of the most commonly occurring disabling conditions, affecting over 364 million adults (Li et al., 2024), and it is widely recognized as the most prevalent and representative form of osteoarthritis (OA). The current clinical treatment faces three major problems. First, pharmacologic therapy is limited; according to Osteoarthritis Research Society International (OARSI) guidelines, non-steroidal anti-inflammatory drugs (NSAIDs) can only provide short-term symptom relief while carrying substantial risks of adverse gastrointestinal and cardiovascular events (Bannuru et al., 2019). Second, intrarticular therapy hits a bottleneck. Corticosteroid injection provides only a brief pain-alleviating experience (<4–6 weeks), while repeated injections accelerate cartilage degradation (McAlindon et al., 2017). Third, although total knee replacement can relieve end-stage pain, it comes with risks such as a long recovery period (3–6 months), prosthesis loosening, and surgical site infection (Jang et al., 2021). Summary, a simple challenge remains: the existing treatments at present offer only symptomatic treatment but not reversal of disease. To escape from this dead end, we need faster development of new, mechanism-based therapies.

Growing evidence points to ferroptosis (cell death caused by iron and lipids builds up as a by-product of this dehydration activity) as a reason for degeneration of chondrocytes in OA. Impairment in Glutathione peroxidase 4 (GPX4) function, which attenuates cellular redox defense, i s also associated with cartilage extracellular matrix (ECM) damage (Miao et al., 2022) implying targeting ferroptosis as a promising new direction for possible interventions in OA pathogenesis. By the same token, the gut-joint axis also involved in OA generation via several known mechanisms (Jeyaraman et al., 2023). Specifically, intestinal dysbiosis results in the loss of the gut barrier integrity that allows lipopolysaccharide (LPS) translocation from bacteria into circulation where LPS acts as a ligand-dependent toll-like receptor 4 (TLR4) stimulator initiating downstream signaling cascades amplifying the systemic inflammatory cascade (Longo et al., 2024). Furthermore, decreased biosynthesis of microbiota-derived short-chain fatty acids (SCFAs) compromises SCFAs’ dual defensive roles against inflammation and chondrocyte degeneration (Han et al., 2025). While ferroptosis and gut dysbiosis have been extensively studied in isolation, growing evidence supports profound molecular crosstalk between these pathways, which synergistically drive OA progression. This review, for the first time, systematically synthesizes the crosstalk mechanisms between gut-joint axis signaling and ferroptosis in OA. Based on the phenomena directly observed in the OA model, the mechanisms verified in other chronic inflammatory/degenerative diseases [such as rheumatoid arthritis (RA)], and reasonable inferences based on the cross-linkages of molecular pathways, we propose a hypothetical framework, suggesting that the “gut microbiota-ferroptosis axis” ma contribute to the progression of OA and explore the therapeutic potential of synergistic intervention measures targeting this axis.

## 2 Ferroptosis in OA: molecular mechanisms and pathological significance

### 2.1 Ferroptosis in OA pathogenesis: emerging connections

Characterized by iron dysregulation and lipid peroxide overload, ferroptosis—originally triggered by the small molecule erastin—was formally defined in 2012 by Dixon’s seminal work (Yang J. et al., 2021; Miao et al., 2022). Emerging evidence positions ferroptosis as a contributing driver to OA pathogenesis. Osteoarthritic joints exhibit dysregulation of core ferroptosis regulators in critical signaling pathways (Ji et al., 2024). Moreover, ferroptotic activation induces overexpression of matrix metalloproteinases (MMPs) and a disintegrin and metalloproteinase with thrombospondin motifs (ADAMTS), which disrupts articular cartilage metabolic homeostasis and potentiates ECM degradation, thereby driving OA progression (Sheng et al., 2025). Therefore, ferroptosis is considered an important target and key breakthrough point for treating OA.

### 2.2 Mechanism of ferroptosis occurrence

#### 2.2.1 Core regulatory pathways

##### 2.2.1.1 System xc^−^/GSH/GPX4 signaling as primary regulatory hub

Ferroptosis regulation is widely recognized as primarily involving the constitutive inhibition of system Xc^−^, which triggers a cascade leading to glutathione (GSH) exhaustion and GPX4 functional impairment. So, this cascade disabled the cell capacity to convert toxic lipid hydroperoxides (L-OOH), such as malondialdehyde (MDA) and 4-hydroxy-trans-2-nonenal, into less-toxic lipid alcohols (L-OH) and culminated in cell death from lipid peroxidation. System Xc^−^ transports cystine (an alternative precursor of GSH) into cells and exports an equivalent number of glutamate out of the cells through solute carrier family 3 member 2 and solute carrier family 7 member 11 (SLC7A11) transporters (Lu et al., 2024; Zhang et al., 2025a). GPX4 normally catalyzes GSH to glutathione disulfide (GSSG) and further reduces L-OOH to L-OH to protect cells against damage by lipid peroxides (LPO) (Liu et al., 2025; Zhang et al., 2025a). In addition to this function, GPX4 regulates the mitogen-activated protein kinase/nuclear factor kappa-light-chain-enhancer of activated B cells (MAPK/NF-κB) signal axis to prevent ECM degradation (Miao et al., 2022; Song C. et al., 2025; Wang D. et al., 2025; Zhou et al., 2025). Reduced GPX4 expression in turn means that this protective system is reduced and thus enhancing susceptibility of chondrocytes to oxidative stress via MAPK/NF-κB signaling (Liu et al., 2024). Thus, summarizing, ferroptosis is basically system Xc^−^ inhibition dependent and driving a process that culminates in the fat-induced final lethal step of the lipid peroxidation and GPX4 play a prominent protective role in it.

##### 2.2.1.2 ACSL4-driven esterification

Acyl-CoA synthetase long-chain family member 4 (ACSL4)-mediated esterification reactions are another milestone of ferroptosis execution (Guo et al., 2022). ACSL4 can catalyze the esterification of PUFAs in phospholipids on cellular membranes and enhance ferroptosis sensitivity to lipid peroxidation (Zhang et al., 2025c). At the molecular level, the molecular basis of this process results from the polyunsaturated double-bond structure of PUFA, which, as a consequence, make them easily prone to peroxidation, giving PUFAs pre-dominant roles in mediating ferroptosis (Wang F. et al., 2025).

ACSL4 catalyzes the reaction of PUFAs to polyunsaturated fatty acid-coenzyme A (PUFA-CoA), and then the PUFA-CoA undergoes esterification by lysophosphatidylcholine acyltransferase 3 (LPCAT3) to become polyunsaturated fatty acid-phospholipids (PUFA-PLs), which are then oxidized by reactive oxygen species (ROS) to cytotoxic phospholipid hydroperoxides (PL-OOH), leading to cell death through membrane modification (Liu et al., 2025; Zhang et al., 2025a; Zou et al., 2025). Critically, the oxidative breakdown of integrated PUFA-PLs affects membrane integrity whereas isolated olyunsaturated fatty acid hydroperoxides (PUFA-OOH) have no membrane-disrupting properties (Liu et al., 2024). Notably, ACSL4-driven lipid peroxidation is significantly potentiated under GPX4 deficiency (Wang R. et al., 2025).

Inhibiting ACSL4 expression reverses the inhibitory effect of interleukin-1β (IL-1β) on collagen II (COL II) and aggrecan mRNA expression in chondrocytes, while alleviating IL-1β’s promoting effect on matrix metalloproteinase 13 (MMP13) mRNA expression (Sheng et al., 2024). Experimental evidence confirms that paeonol confers protection against ferroptosis and dysregulated ECM metabolism in chondrocytes through suppression of ACSL4 expression and activity (Cao et al., 2025). Furthermore, thiazolidinedione derivatives (e.g., rosiglitazone and pioglitazone) demonstrate selective inhibition of ACSL4, consequently attenuating ferroptosis progression (Sheng et al., 2024). These findings substantiate the therapeutic merit of ACSL4 pathway modulation in OA intervention.

##### 2.2.1.3 Mechanism of SLC7A11 and p53 in the regulation of ferroptosis

Suppression of SLC7A11, a key component of system Xc^−^, induces intracellular GSH depletion, subsequently initiating ferroptosis (Guo et al., 2022; Gu et al., 2025; Wang F. et al., 2025). Studies demonstrate that SLC7A11 activates mechanistic target of rapamycin complex 1 signaling, which phosphorylates 4E-binding protein 1 to enhance GPX4 protein translation; this molecular cascade ultimately strengthens cellular antioxidant defenses (Zhang et al., 2024a).

Beyond its central role in apoptotic regulation, the tumor suppressor protein 53 (p53) critically contributes to ferroptosis by suppressing SLC7A11 expression; this inhibition reduces intracellular cystine availability, resulting in GPX4 functional impairment and consequent ferroptosis execution (Guo et al., 2022; Zhang et al., 2025c). Furthermore, recent studies have indicated that p53 may regulate the abnormal differentiation of osteoclasts through the p53/forkhead box protein O3 (FOXO3) signaling pathway, thereby modulating ferroptosis in chondrocytes (Zhao et al., 2025). We propose that the tumor suppressor p53 may play a role in the pathophysiological process of OA through a “bidirectional regulation of cell fate” mechanism. On one hand, p53 reduces cystine uptake by inhibiting SLC7A11 expression, leading to impaired function of GPX4, thereby inducing ferroptosis in lesioned chondrocytes to eliminate damaged cells. On the other hand, it may suppress pathological osteoclast maturation via the p53/FOXO3 signaling pathway and enhance the antioxidant defense system to shield healthy chondrocytes against ferroptosis. This dual role suggests that p53 may act as a promising therapeutic target in OA therapy, achieving the synergistic effect of “eliminating lesioned cells while protecting normal tissues” by precisely regulating the balance between apoptosis and ferroptosis in different cell populations. Future research can focus on using specific gene knockout/knock-in animal models and combining single-cell sequencing technology to clarify the spatiotemporal expression patterns and functional differences of p53 in different cell types (such as chondrocytes, synoviocytes, and osteoclasts) and different stages of OA. Through molecular interaction experiments (such as Co-IP and dual-luciferase reporter gene assays), the regulatory mechanisms between p53 and key target molecules such as SLC7A11 and FOXO3 can be analyzed to elucidate the molecular switch of its bidirectional effects. Based on the p53 regulatory network, small molecule modulators or gene editing tools can be designed, and their therapeutic effects on cartilage protection and inflammation alleviation can be validated in OA animal models. Additionally, their synergistic effects with existing clinical drugs can be explored.

#### 2.2.2 Key driving factors

##### 2.2.2.1 Iron overaccumulation

The core driving factor of ferroptosis is the excessive accumulation of iron, and this process is mainly regulated by iron homeostasis regulators such as transferrin receptor (TFR), ferritin, and ferroportin (FPN) (Zhang et al., 2025c). Under physiological conditions, cells achieve the transmembrane transport of the ferric ion (Fe^3+^)-transferrin complex through the endocytic pathway mediated by TFR1; the acidic microenvironment in the endosome will promote the dissociation of Fe^3+^ from transferrin; Fe^3+^ is catalytically reduced to ferrous ion (Fe^2+^) by six-transmembrane epithelial antigen of the prostate 3 (STEAP3), and then enters the cytoplasm through divalent metal transporter 1 (DMT1) to form a labile iron pool (LIP); cells can use ferritin (FTN) polymers to chelate and store excess Fe^2+^, and FPN completes the efflux of Fe^2+^ regulated by hepcidin (Lu et al., 2024; Liu et al., 2025; Zhang et al., 2025a).

When there is excessive accumulation of LIP in chondrocytes, the overloaded Fe^2+^ catalyzes the explosive generation of ROS through the iron-dependent Fenton reaction (Yang et al., 2024). The pathological accumulation of ROS first leads to mitochondrial dysfunction, manifested as disruption of the electron transport chain and collapse of membrane potential; subsequently, it causes oxidative damage to lipids, proteins, and DNA through a free radical chain reaction (Zhang R. et al., 2025; Zhang et al., 2025a). Ultimately, this results in excessive accumulation of LPO, exceeding the threshold of cellular repair capacity (Liu et al., 2025), thereby activating the ferroptosis execution program in osteoarthritic chondrocytes.

Under iron deficiency conditions, nuclear receptor coactivator 4 (NCOA4) activates the ferritinophagy pathway (mainly targeting ferritin heavy chain 1) to dynamically release stored iron (Lu et al., 2024; Zhang et al., 2025a). NCOA4, as a key receptor for ferritinophagy, can specifically bind to the conserved surface arginine residues of the ferritin heavy chain at its C-terminus, promoting iron release during autophagosome formation and autolysosome maturation (Liu et al., 2025). When NCOA4 is upregulated, it can increase the intracellular iron pool by mediating ferritinophagy, significantly promoting lipid peroxidation and ferroptosis (Lu et al., 2024).

##### 2.2.2.2 Mechanical stimulation

Mechanical stimulation is tightly linked to ferroptosis in chondrocytes derived from OA joints. Specifically, the mechanically sensitive ion channel piezo-type mechanosensitive ion channel component 1 (PIEZO1), as a key sensor of mechanical stress, activates calcium ion (Ca^2+^) influx; this contributes to mitochondrial ROS accumulation and abnormal autophagic activity, which may drive ferroptosis processes (Liu et al., 2025; Zhang et al., 2025a). Under the stimulation of high mechanical stress, activation of the PIEZO1 protein leads to a decrease in the intracellular GSH level of chondrocytes, a reduction in the function and expression of GPX4, and a significant increase in the intracellular calcium ion concentration, ultimately inducing ferroptosis of chondrocytes; this process is characterized by enhanced oxidative stress, mitochondrial structural and functional disorders, and dysregulation between the synthesis and catabolism of chondrocytes (Liu et al., 2024).

Research shows that inhibiting the mechanically sensitive ion channel PIEZO1 can upregulate the expression of GPX4 by blocking calcium ion influx, effectively inhibit ferroptosis of chondrocytes, and significantly alleviate the pathological damage of OA (Liu et al., 2024). Meanwhile, studies have shown that GPX4 may be a key downstream target of PIEZO1 (Du et al., 2025). However, the exact signaling pathway involved has yet to be clarified in future studies.

Experiments have shown that the specific inhibitor of PIEZO1 can significantly reduce iron overload, inhibit mitochondrial oxidative stress, and block the characteristic changes of ferroptosis induced by mechanical stress (Liu et al., 2025). This highlights its crucial regulatory role in maintaining cartilage homeostasis. Meanwhile, the mechanical signal-PIEZO1-ferroptosis molecular axis has been confirmed to be involved in the pathological processes of cartilage degeneration and aging (Liu et al., 2025). As the core molecular hub between the mechanical microenvironment and the cellular ferroptosis program, i t might serve as a source of new potential targets for OA therapy.

### 2.3 Ferroptosis defense pathways independent of the GPX4 system

#### 2.3.1 FSP1/NADPH/CoQ₁₀pathway

The ferroptosis suppressor protein 1/nicotinamide adenine dinucleotide phosphate/coenzyme Q₁₀ (FSP1/NADPH/CoQ₁₀) regulation channel is an important defense path independent of GPX4 system, which plays very important function (Zhang et al., 2024a). FSP1 regenerates CoQ₁₀ based on NADPH; the action effect of CoQ₁₀ as a oxidation substrated replaces PUFA-PL and blocks lipid peroxidation damage (Liu et al., 2024); but can also scavenge LPO through free radical capture lipophilicity ability of ubiquinol coenzyme Q₁₀ (CoQ₁₀H₂) (Zhang et al., 2025a). In addition, CoQ₁₀H₂ could not only indirectly promote the the fat-soluble antioxidant α-tocopherol to regenerate but also cooperate with it to scavenge free radicals and inhibit ferroptosis (Wang Z. et al., 2024; Zhang et al., 2024a). In addition to its basic antioxidant function, FSP1 can also induce inhibition of ferroptosis from another aspect–membrane repair action via endosomal sorting complex required for transport -III (Zhang et al., 2024a), yet these results need deeper research. Relevant studies showed that high mechanical load conditions supplementing ferroptosis bypass regulation factor FSP1 into chondrocytes would obviously alleviate the process and damages induced by chondrocyte ferroruptosis (Du et al., 2025). It proved once again there was close correlation between them.

#### 2.3.2 GCH1/BH4 pathway

**Figure 1:**
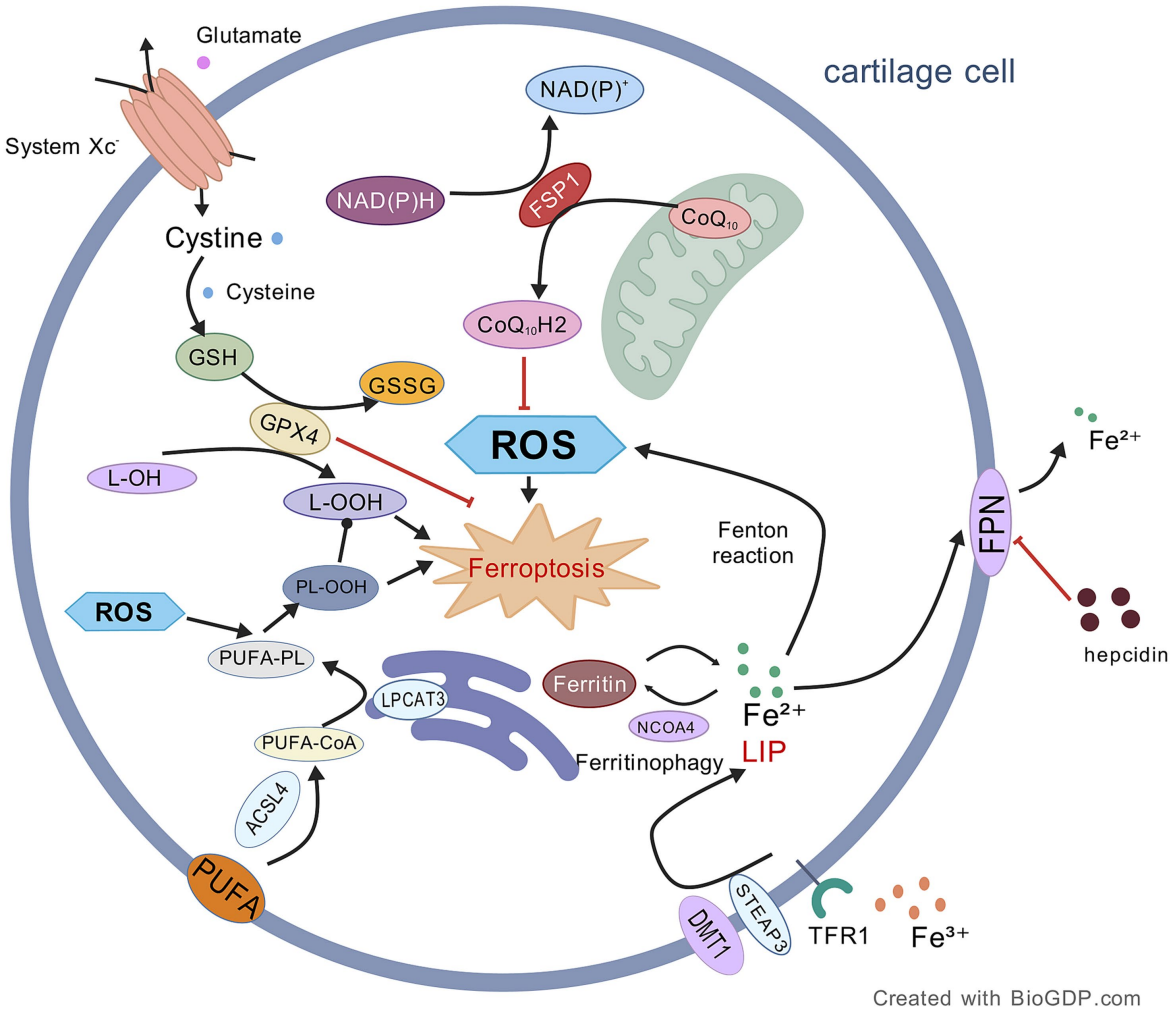
Mechanism of ferroptosis in OA chondrocytes. This figure illustrates the core mechanisms driving ferroptosis, primarily mediated by membrane phospholipid peroxidation (facilitated by ACSL4/LPCAT3-dependent oxidation of PUFA-containing phospholipids) and iron overload (involving Fe^2+^ release via NCOA4-mediated ferritinophagy). These pro-ferroptotic processes are counterbalanced by key antioxidant systems, namely the GPX4/GSH and FSP1/NADPH/CoQ₁₀ axes. Consequently, inactivation of GPX4 or depletion of GSH serves as a primary trigger for ferroptosis induction. Black lines: promotion of processes/mechanisms. Red lines: inhibition of processes. Solid lines: experimentally proven relationships. Dashed lines: proposed relationships. Figures all created with BioGDP.com (Jiang et al., 2025).

The GTP cyclohydrolase 1/tetrahydrobiopterin (GCH1/BH4) pathway is another ferroptosis inhibition mechanism independent GPX4 system (Liu et al., 2024). As a strong antioxidant, BH4 maintains cell redox balance due to its powerful redox activity; at the same time, researches show that GCH1 has specific competitive antagonism with ferroptosis (Wang Z. et al., 2024). GCH1’s anti-lipid peroxide action is accomplished by catalyzing the generation of BH4 acting like CoQ₁₀, but it also enhanced the mechanism of resistance to lipoperoxidation by scavenge PUFA-PLs and recycle depleted CoQ₁₀ (Liu et al., 2024). In recent works, the study demonstrated that GCH1/ BH4 pathic enzyme dihydrofolate reductase (DHFR) could inhibit ferroptosis by generating BH4, so decrease in DHFR activity reduces BH4 content to enhance lipid peroxidation and sensitizes cells to ferroptosis (Lu et al., 2024), but the detail process is still unknown (Figure 1).

## 3 The role of the gut-joint axis in OA: microbiota, metabolites, and regulatory networks

### 3.1 Concept of the gut-joint axis

The gut microbiome, a diverse community of microorganisms predominantly composed of bacteria and encompassing archaea, fungi, and viruses, colonizes the gastrointestinal tract; it exerts multifaceted effects on host health, including maintaining intestinal barrier integrity, regulating immune responses, and modulating metabolic processes (Meléndez-Oliva et al., 2025). Emerging evidence suggests the existence of a “gut-joint axis,” wherein the gut microbiome influences the pathogenesis of joint diseases via discrete mechanisms. This mechanism likely originates from microbial-derived bioactive molecules that enter systemic circulation to regulate physiological processes in distal organs (Meléndez-Oliva et al., 2025). Specifically, dysbiosis of gut microbiota compromises intestinal barrier function, leading to high intestinal permeability; this allows translocation of dietary antigens, intact bacteria and bacteria toxins into systemic circulation, as well as microbiota metabolites (such as LPS and peptidoglycan) in smaller molecules (Han et al., 2025; He M. et al., 2025). Systemic and local levels of these pro-inflammatory metabolites—including LPS, peptidoglycan and fatty acids—are elevated and cause damage in joint tissues by bringing about metabolic endotoxaemia, macrophage invasion and chronic low-grade inflammation (Han et al., 2025; Hao et al., 2025; He M. et al., 2025). From this inflammatory reaction disseminates to other tissue in the joint hematogenously; a resident pool of immune cells (for example, macrophages and synovial fibroblasts) is stimulated; this enhances the elaboration of proinflammatory cytokines and enzymes for ECM degradation and leads to cartilage damage (Han et al., 2025). Alternatively, impaired intestinal barrier function allows microbial translocation; these translocated elements spread through the vasculature and establish foci of localized dysbiosis that provoke local inflammatory response in joint tissue (He M. et al., 2025). Ultimately, the gut microbiota can now be regarded as a crucial “distal regulator” of OA pathogenesis in many ways including regulation of the intestinal barrier function, effect on immune-inflammatory mechanisms and regulation of host metabolic disorders. Thus, this also provides a new paradigm to understand the complex pathological network in OA and offers a theoretical guidance for gut microbiota modulation approaches for the OA prevention or OA therapeutic strategy such as probiotic approach, fecal microbiota transplantation (FMT) or detoxification of certain metabolites.

### 3.2 Gut microbiota alterations in OA

#### 3.2.1 Gut microbial dysbiosis in OA

Recent years have seen many novel studies unveiling the important role of gut microbiota dysbiosis in OA pathogenesis, with strong evidence for the “gut-joint axis” hypothesis of OA. Gross changes were found in the gut microbiota in both OA patients and murine OA models (Han et al., 2025), so dysbiosis of the gut microbiota is useful for leading to OA pathogenesis. Much higher levels of Proteobacteria are also present in the knee joints of OA patients than in the control samples without OA (Heckmann et al., 2025). Moreover, increased intestinal *Streptococcus* abundance positively associates with worsening knee symptoms (Han et al., 2025; Hao et al., 2025; Heckmann et al., 2025), whereas greater *Akkermansia* and *Bacteroides* abundance demonstrates a significant inverse association with OA risk (Kurz et al., 2025). Additionally, OA severity and inflammatory biomarker levels correlate with increased *Fusobacterium* abundance, elevated *Faecalibacterium prausnitzii* levels, and decreased *Ruminococcaceae* family representation in the gut microbiota (Gaspar et al., 2025; Han et al., 2025; Heckmann et al., 2025). Collectively, the gut microbiota dysbiosis mediated by these specific bacterial populations may jointly promote the occurrence and progression of OA through multiple pathways such as regulating the host’s immune-inflammatory response, metabolic disorders, and oxidative stress, providing novel targets and strategies based on gut microbiota regulation for the prevention and treatment of OA.

#### 3.2.2 Phylum Bacteroidetes

As a core phylum in the human gut microbiota, Bacteroidetes maintains intestinal barrier integrity, regulates immune responses, and facilitates pathogen exclusion; consequently, reduced Bacteroidetes abundance correlates with microbial dysbiosis and impaired mucosal barrier function (Wang Y. et al., 2025). The research found that when the abundance of *Clostridium* spp. increases, it can significantly weaken the colonization potential of *Bacteroides* spp. in the intestine through niche competition (Ji et al., 2023). The significant reduction in *Bacteroides* abundance observed during OA onset supports the protective role of Bacteroides in OA pathogenesis, potentially mediated through intestinal homeostasis maintenance (Kurz et al., 2025). Paradoxically, in advanced KOA with osteoporosis comorbidity, Bacteroidetes abundance positively correlates with disease progression and enriched in murine intestinal inflammation models (Longo et al., 2024). This phenomenon may be specifically attributed to *Bacteroides fragilis* enrichment, which compromises intestinal barrier integrity through mucin degradation-mediated disruption of the mucosal layer (Ji et al., 2023; Longo et al., 2024). This suggests that the “double-edged sword” effect of Bacteroidetes may depend on its specific strain composition and the pathophysiological state of the host. Therefore, in-depth analysis of the exact regulatory network of Bacteroidetes in the pathogenesis of OA through the gut-joint axis, and clarification of the functional specificity of different Bacteroides strains and their interactions with host metabolism, immune and inflammatory pathways, are of great theoretical and clinical significance for accurately understanding the mechanism of the influence of gut microbiota on OA and developing probiotic intervention strategies targeting specific strains.

#### 3.2.3 Phylum Proteobacteria

As a key component of the gut microbiome predominantly composed of Gram-negative bacteria, increased Proteobacteria abundance is closely associated with epithelial barrier dysfunction and elevated intestinal permeability (Heckmann et al., 2025). Intestinal barrier compromise facilitates Proteobacteria translocation into systemic circulation, coinciding with elevated pro-inflammatory mediators, heightening systemic inflammation and exacerbating OA severity; conversely, reduced Proteobacteria abundance correlates with significant OA symptom amelioration (Longo et al., 2024). Therefore, the imbalance of the abundance of Proteobacteria may be one of the mechanisms by which the disorder of the gut-joint axis drives the progression of OA. This suggests that Proteobacteria may become a potential target for intervening in OA by regulating the gut-joint axis. In the future, in-depth analysis of the molecular mechanisms by which it induces gut microbiota dysbiosis (such as LPS release, activation of inflammatory signaling pathways, etc.) may offer a significant theoretical basis to support the development of OA therapeutic.

#### 3.2.4 Akkermansia muciniphila

*Akkermansia muciniphila*, a Gram-negative anaerobic bacterium, specifically colonizes the intestinal mucus layer, critically modulating the microbiota-host inflammatory response-intestinal barrier axis; reduced abundance of this bacterium significantly correlates with diminished mucus layer thickness and heightened pro-inflammatory states (Ji et al., 2023). Critically, *Akkermansia muciniphila* enhances intestinal barrier function at optimal abundance but promotes mucosal degradation when exceeding homeostatic thresholds; this dysbiotic state facilitates systemic translocation of toxins (e.g., LPS), consequently exacerbating OA pathology (Kurz et al., 2025). Taken together, *Akkermansia muciniphila* may exert a “double-edged sword” effect on the homeostasis of the gut-joint axis through changes in its abundance: at moderate levels, it enhances intestinal barrier function and inhibits inflammation, while dysbiosis (either excessive or insufficient abundance) exacerbates the pathological progression of OA by disrupting mucus layer integrity and promoting toxin translocation. Future studies should prioritize defining the precise abundance threshold of *Akkermansia muciniphila* that balances its beneficial and deleterious effects on the intestinal barrier, investigating the molecular mediators (e.g., specific metabolites or surface proteins) that underpin its dual regulatory role, and developing targeted interventions (e.g., precision probiotics or microbiota modulators) to sustain its homeostatic levels—with the goal of ultimately mitigating OA progression via the gut-joint axis.

## 4 Hypothesis of the “gut microbiota-ferroptosis axis” in OA pathogenesis

### 4.1 Gut microbiota and abnormal iron metabolism synergistically promote joint ferroptosis

#### 4.1.1 Gut microbiota promotes ferroptosis via hepcidin-FPN1 axis and FTN-mediated iron overload

The gut microbiota modify systemic iron homeostasis through the modulating ability of microbiota to regulate the synthesis of hepatic hepcidin, an effect the subject of intense investigation. For iron metabolism, hepcidin acts on FPN and promotes its ubiquitination, endocytosis and degradation or inhibition of its channel activity to keep the intracellular iron in storage; hepcidin also downregulates its synthesis through a negative feedback loop depending on circulating iron concentration to prevent iron overload in the body (Yao and Li, 2023). However, dysbiosis of the gut microbiota would likely alter hepcidin secretion and cause iron loading in joint cells, promoting ferroptosis in OA. Future efforts could consider studying the progression of “hepcidin-FPN1 axis” dysregulation over the course of OA. Such studies will help distinguish whether it is gut microbiota dysbiosis-induced iron overload that affects chondrocyte ferroptosis and how, potentially, via lipid peroxidation or GSH loss. Furthermore, the specific microbial taxa and their metabolites that control hepatic hepcidin production need to be identified and signaling by SCFAs, bile acid metabolites (BAM), or tryptophan metabolites examined in particular as to which of these are key signaling molecules in this axis.

FTN is a master regulator of intestinal iron absorption and systemic iron homeostasis. Intestine-specific FTN-deficiency leads to marked increases in intestinal iron absorption and inducing iron overload, and an unidentified novel microbial metabolite regulates systemic FTN homeostasis (Das et al., 2020). Gut microbiota can also affect host iron-absorbing efficiency through siderophore secretion, chelation of iron and the control of the expression of host iron-transporter molecules (Mao et al., 2024). Most specifically, some bacterial metabolites enhance the host iron transport system, and others are chelators that interfere with iron absorption (Jian and Wei, 2025). Notably, gut microbiota metabolites suppress intestinal epithelial Hypoxia-inducible factor-2 alpha (HIF-2α) activity and reduce the expression of FTN and, thus, regulate the host iron absorption pathway (Mao et al., 2024). Indeed, experimental data confirm that metabolites 1,3-diaminopropane and reuterin inhibit the heterodimerization of HIF-2α with the aryl hydrocarbon receptor nuclear translocator (ARNT) and markedly inhibit tissue iron accumulation in overload models (Das et al., 2020). Additionally, LPS boosts serum iron through activation of Caspase-11/Gasdermin D pathway (Liu et al., 2022b). Together, these results mean that the metabolites of gut microbiota disturb regulation of joint FTN and homeostasis of iron, thereby may inducing iron overload that triggers ferroptosis and accelerate the development of OA. In the future, there are several priorities that should be addressed. First, isolate newly identified microbial metabolites that modulate systemic FTN homeostasis, establish their molecular targets and signaling cascades in intestinal epithelial cells—in particular, how they interact with the dimerization of HIF-2α/ARNT. Second, summarize the strain-specific impacts of the gut microbiota on bacterial siderophore synthesis, iron sequestration, and the expression of host iron transporters to provide a basis for accurate probiotic regulation. Third, elucidate the synergistic and/or antagonistic effects of the metabolites (e.g., 1,3-diaminopropane) on iron release during signaling pathways like HIF-2α/FTN, yet verify their regulation of joint ferroptosis. Fourth, apply *in vivo* models to explore the therapeutic effect of microbiota-targeting treatments (such as metabolite supplements and microbial engineering) to restore iron homeostasis and ameliorate the OA progression and put forward evidence for clinical trials.

#### 4.1.2 Iron overload promotes joint ferroptosis by disrupting gut barrier

Imbalanced host iron homeostasis remodels the structure of the gut microbiota, which can be manifested as changes in alpha-diversity, alterations in taxonomy composition and community dominance (Mao et al., 2024). Furthermore, iron overload triggers an oxidative stress cascade that disrupts intestinal immune homeostasis and barrier function in mammalian hosts, leading to gut microbiota dysbiosis characterized by decreased abundance of probiotics (Gu et al., 2023; Mao et al., 2024). This process activates the colonic ferroptosis pathway and induces systemic inflammation (Gu et al., 2023). Concurrently, reduced levels of anti-inflammatory metabolites exacerbate this systemic inflammation (Long et al., 2024). Ultimately, iron overload may promote the progression of OA through a cascade mechanism that disrupts intestinal homeostasis and exacerbates systemic inflammation.

Many anaerobic bacteria that are normal (such as *Bifidobacterium*) are close to the intestinal epithelial cells to form a biofilm protection layer, which can compete with colonization and immune defense (Ji et al., 2023). Iron overload, however, significantly reduces *Bifidobacterium* abundance, a disturbance potentially linked to gut microbiota dysbiosis mediated by intestinal ferroptosis; exogenous *Bifidobacterium* supplementation can effectively counteract these alterations and alleviate intestinal inflammatory responses (Gu et al., 2023). Specifically, soluble lipoproteins produced by *Bifidobacterium longum* markedly inhibited OA pathological process via microbiota diversity adjustment (Sane et al., 2023). Therefore, iron overload and its induced depletion of *Bifidobacterium*, along with intestinal dysfunction, form a synergistic effect on the “gut-joint axis.” By exacerbating local ferroptosis and systemic inflammation, they may synergistically contribute to the pathogenesis and progression of OA. The complex interaction relationship between iron metabolism disorders and changes in the abundance of gut microbiota (especially *Bifidobacterium*) revealed in this section provides evidence for clarifying the molecular mechanism of the hypothetical pathological axis of “iron overload-disruption of gut homeostasis-ferroptosis in OA,” and suggests that targeted regulation of the iron-microbiota interaction may become a potential new strategy for the prevention and treatment of OA.

### 4.2 Potential impact of gut microbiota on ferroptosis in OA

#### 4.2.1 Gut dysbiosis promotes lipid peroxidation and ferroptosis in OA

In patients with OA, the significant dysregulation of the gut microbiota not only manifests as a disorder in the community structure but also profoundly affects the pathological process of OA by precisely regulating the key hub genes in the host lipid metabolism network. Studies have shown that significant gut microbiota dysbiosis in OA patients modulates key lipid metabolites, contributing to OA progression. This occurs via the lipid metabolism hub gene Phospholipase A2 Group IIA in homoeostatic chondrocytes, a Phospholipase A2 enzyme that promotes OA pathology by increasing arachidonic acid release and activating downstream pro-inflammatory pathways (Wang Y. et al., 2025). Subsequently, arachidonic acid is esterified into phosphatidylethanolamine-arachidonic acid under ACSL4 catalysis, promoting the accumulation of LPO that drive ferroptosis in OA (Cao et al., 2025). Therefore, gut microbiota dysbiosis may promote OA ferroptosis progression through alterations in microbial composition and metabolite profiles, which enhance lipid peroxidation. It suggests that targeting the gut microbiota structure to correct lipid metabolic disorders and inhibit LPO-induced ferroptosis may become a potential therapeutic strategy to block the progression of OA.

#### 4.2.2 Potential impact of specific gut microbiota on ferroptosis in OA

##### 4.2.2.1 *Prevotella copri:* contradiction between human OA enrichment and mouse model protection

Recent research on OA has revealed that the endogenous bacterium *Prevotella copri (P. copri)* alleviates ferroptosis in mouse calvaria-derived pre-osteoblastic cell line, subclone E1 (MC3T3-E1) osteoblasts by metabolizing caffeine into paraxanthine; this metabolite regulates oxidative stress pathways and suppresses lipid peroxidation (Li et al., 2025). Experimental evidence further demonstrates that *P. copri* intervention, mediated through gut barrier repair, significantly restores bone homeostasis—including enhanced bone density and bone remodeling balance—and promotes osteoblast ferroptosis resistance in both *in vitro* cellular models (e.g., MC3T3-E1 cultures) and *in vivo* animal models (e.g., OA mice) (Li et al., 2025). This study confirms an important role of *P. copri* in inhibiting ferroptosis and repairing the gut barrier in the OA pathogenesis process and provides direct evidence for the relation of gut-joint axis and ferroptosis in OA. However, it is interesting that another study demonstrates that *Prevotella* were enriched at the genus level in OA patients (Han et al., 2025). This seemingly paradoxical phenomenon—where *P. copri* is enriched in human OA patients yet beneficial in mouse models—urgently requires intensive investigation in multiple aspects, such as strain-specific differences (e.g., metabolic function distinctions between pathogenic and beneficial strains), dynamic regulation of the host immune microenvironment (e.g., the effect of inflammatory status on the microbiota–host interaction), and the effect of environment in influencing the effects of metabolites (for example, the effect of regulation of gut niches and availability of nutrient substrate for the production of bioactive entities such as paraxanthine). Future studies should strive to define at precise functional levels (the species/strain-level degree of functional heterogeneity) in the genus *P. copri* through deep metagenomic sequencing and *in vivo* experiments using establishment of germ-free mice with different *P. copri* strains. These efforts will enable the clarity of metabolic nature, immunologic effects of the specific strains, their major differential modes of impact on the “gut microbiota-ferroptosis axis,” and an understanding of the underlying scientific essence for this contradiction, thereby offering a potential basis for prevention and treatment of OA with precise microbiota regulation.

##### 4.2.2.2 Reduced *Lactobacillus* in OA: iron metabolism regulation and probiotic potential

For patients with KOA, the abundance of *Lactobacillus* significantly decreased (Gaspar et al., 2025). Its lower abundance correlates with such a phenomenon that iron excess reduced *Lactobacillus* abundance in the gut (Gu et al., 2023). The results indicated that *Lactobacillus* might contribute to pathogenesis of OA by involving iron metabolism and ferroptosis. Specifically, it was found that Latilactobacillus sakei LB-P12 slowed down OA progression by targeting NF-κB/HIF-2α pathway activation in macrophages and chondrocytes to suppress inflammation and inhibit cartilage ECM degradation (Song M. et al., 2025). In parallel, the HIF-2α (Lu et al., 2024) and NF-κB signaling (Huo et al., 2024) pathway promoted OA-derived ferroptosis. Moreover, NF-κB pathway activation impaired the GSH/GPX4 axis by triggering HIF-2α generation (Zhang et al., 2024c). Existing studies simply showed that the reduced gut *Lactobacillus* abundance in OA patients was associated with iron overload and chondrocyte ferroptosis. Future studies need to concentrate on three points: deepening insights about mechanisms; verifying whether it involves causality; and translating it in clinical applications. Specifically, germ-free mouse models or FMT can be adopted to confirm a causal chain that iron overload mediates a reduction of gut *Lactobacillus*, which in turn aggravates the development of OA. Besides, it is important to elaborate out the core driving mechanisms of the “gut microbiota-ferroptosis axis” to facilitate our understanding. Another task to be performed is to identify the functional effector molecules of the driver *Latilactobacillus sakei LB-P12* (e.g., SCFAs and bacterial extracellular vesicles) and their pathways for crossing the intestinal epithelial barrier and targeting joint macrophages and chondrocytes. And finally, further screening of other strains of Lactobacillus and genetic engineering of specific *Lactobacillus* strains should help in understanding and exploring their broader probiotic therapeutic role. These studies will further deepen our understanding of “gut microbiota-iron metabolism-ferroptosis” regulatory network and also offer theoretical guidance and a translational scaffold for the OA precision-targeted therapy.

##### 4.2.2.3 Context-dependent dual role of *Escherichia coli* in ferroptosis regulation

One study indicates that *Escherichia coli (E. coli)* causes ferroptotic cell death of erythrocytes by inducing the Fenton reaction to release ROS and increase internal iron levels (Yang M. et al., 2021). This finding implies a possible role for *E. coli* in activation of ferroptosis during the pathogenesis of OA, consistent with an observed increase in the abundance of *E. coli* in patients with OA (Gaspar et al., 2025). Paradoxically, Surprisingly, other studies, however, reveal that *E. coli* may protect their cells against ferroptosis by assaying Fe^2+^ to deplete the substrates for the Fenton reaction and catalytically decomposing hydrogen peroxide (H₂O₂), ultimately suppressing the production of hydroxyl radical (•OH) (Long et al., 2024). Such contrasting effects clearly demonstrate that the regulatory mechanisms and pathological roles of *E. coli* in OA progression may rely on, for example, the level of Fe^2+^ ions or the numbers of bacteria. Future study may focus on the following priorities on the role of *E. coli* in OA-related ferroptosis. First, explore the triggering conditions (e.g., iron ion content, bacterial content) and molecular factors for the two opposite roles of *E. coli*. Second, investigate the indirect regulation mechanism by which *E. coli* protects/damages chondrocyte ferroptosis over the gut-joint axis. Third, investigate a clinical positive correlation between abundance of *E. coli*, abundance of ferroptosis-related factors (e.g., GPX4, ACSL4) and OA severity in OA patients. Fourth, provide therapeutic options to *E. coli* or its iron metabolism target (e.g., probiotic therapy, iron chelator, etc.) and examine the therapeutic efficiency of slowing onset of OA progression. These studies will provide essential guidance for resolving the conflicting role of *E. coli* in regulating ferroptosis of OA and delineating gut microbiota-mediated etiopathogenesis of the disease.

### 4.3 Potential effects of gut metabolites on ferroptosis in OA

#### 4.3.1 Gut metabolites inhibiting ferroptosis mechanisms

##### 4.3.1.1 Tryptophan metabolites suppress ferroptosis via antioxidant mechanisms

Tryptophan metabolites serve as pivotal mediators in host-microbiota interactions and are closely linked to systemic inflammatory and immune processes (Gaspar et al., 2025). Notably, serotonin (5-HT) and 3-hydroxyanthranilic acid (3-HAA) function as radical-scavenging antioxidants that suppress ferroptosis by attenuating lipid peroxidation (Long et al., 2024). Clinical evidence from hand OA studies reveals significant alterations in gut microbiome-derived tryptophan metabolites: serum levels of 5-hydroxyindoleacetic acid (5-HIAA) and 5-hydroxytryptophan (5-HTP) are elevated, whereas 3-HAA is reduced in symptomatic patients (Wei et al., 2023). This metabolic shift aligns with ferroptosis research wherein 5-HT and 3-HAA promote tumor cell resistance to ferroptosis. Intriguingly, despite structural similarity to 5-HT, neither 5-HTP nor 5-HIAA confers protection against ferroptosis-inducing stimuli (e.g., RAS-selective lethal 3 exposure or GPX4 inactivation) (Liu et al., 2023). In summary, these findings suggest that the branches of the tryptophan metabolic pathway (the kynurenine pathway and the 5-HT pathway) may be the mediating links through which the “gut-joint axis” regulates ferroptosis in OA. Meanwhile, 5-HT and 3-HAA may act as endogenous ferroptosis antagonists, participating in the pathological process of OA by regulating the redox microenvironment of chondrocytes. In the future, in-depth elucidation of the regulatory rules of gut microbiota on tryptophan metabolic shunting and the interaction mechanisms between tryptophan metabolites and core regulatory nodes of ferroptosis, such as host iron homeostasis and GPX4 activity, is not only expected to reveal the regulatory effects of non-cysteine amino acids on ferroptosis, but also provide new theoretical support for the development of precise intervention strategies for OA based on gut microbiota metabolites.

##### 4.3.1.2 Capsiate inhibits ferroptosis through TRPV1/GPX4 and HIF-1α signaling

Capsiate (CAT), a key metabolite of the gut microbiota, exhibits significantly reduced levels in patients with OA (Guan et al., 2023). Previous studies established that CAT inhibits ferroptosis by activating transient receptor potential vanilloid type 1 (TRPV1), thereby upregulating GPX4 expression and ameliorating intestinal ischemia–reperfusion injury as well as organoid hypoxia-reoxygenation injury (Deng et al., 2021). Recent research further demonstrates that CAT suppresses Hypoxia-inducible factor-2 alpha (HIF-1α) expression via activation of solute carrier family 2 member 1 (SLC2A1), effectively reducing ferroptosis-dependent chondrocyte damage and mitigating OA progression (Guan et al., 2023). This directly demonstrates the protective effect of CAT against ferroptosis in OA chondrocytes. However, whether it exerts synergistic regulation via the activation of the TRPV1/GPX4 and SLC2A1/HIF-1α dual pathways remains to be further elucidated. To address this gap, future studies should integrate cryo-electron microscopy and molecular dynamics simulation to elucidate the binding sites and conformational changes of CAT with TRPV1/SLC2A1, as well as the molecular switch mechanism governing the synergistic regulation of the dual pathways. Moreover, using conditional knockout mouse models (e.g., chondrocyte-specific Trpv1^−^/^−^ or Slc2a1^−^/^−^), the protective effects of CAT on ferroptosis and OA progression *in vivo* need to be verified, combined with spatial metabolomics to analyze dynamic changes in lipid peroxidation products and antioxidant molecules in joint tissues following CAT intervention. Additionally, clinical samples should be employed to validate the correlation between serum CAT levels and markers of ferroptosis in articular cartilage (e.g., GPX4 activity) as well as disease severity in OA patients. Finally, it is crucial to explore the key enzymes involved in CAT production by gut microbiota metabolism and their regulatory factors, evaluate the therapeutic potential of engineered probiotics (e.g., CAT-synthesizing strains) or exogenous CAT delivery systems (e.g., cartilage-targeted nanocarriers) for OA, and provide multi-level evidence for CAT-targeted OA therapy based on the gut-joint axis.

##### 4.3.1.3 Creatine inhibits ferroptosis by regulating lipid metabolism and iron homeostasis

Creatine (Cr), an arginine derivative produced by gut bacteria (notably *Bifidobacterium pseudolongum*), is a metabolite that has been proven to effectively inhibit ferroptosis. Specifically, under energy stress, intestinal *Bifidobacterium pseudolongum*-upregulated Cr production activates the AMP-activated protein kinase (AMPK) pathway, suppressing ionizing radiation-induced PUFA synthesis and thereby counteracting ionizing radiation-associated ferroptosis progression (He Y. et al., 2025). Moreover, Cr competitively binds the active site of cellular prion protein, inhibiting the conversion of Fe^3+^ to Fe^2+^ and reducing cellular iron uptake; this mechanism enhances ectopic endometrial stromal cells resistance to ferroptosis (Chen et al., 2024). Further, Mendelian randomization analysis about OA provides additional population-based evidence that serum Cr levels are significantly negatively correlated with low grip strength among people aged 60 and over (Hong et al., 2024), indirectly suggesting its role in improving metabolic homeostasis. Therefore, its inhibitory effect on ferroptosis associated with OA may be achieved by regulating both iron metabolism and lipid metabolism pathways. Given the characteristics of ferroptosis in OA chondrocytes (manifested as iron overload and exacerbated lipid peroxidation), the dual pathways of Cr precisely target these two key driving factors. Therefore, Cr may become a potential intervention target for ferroptosis in OA through the “gut microbiota-Cr-iron/lipid metabolism” axis.

#### 4.3.2 Gut metabolites promoting ferroptosis mechanisms

##### 4.3.2.1 LPS mediates the gut-joint axis to induce ferroptosis in OA

LPS is widely recognized as a mediator of OA pathology via the gut-joint axis. Moreover, LPS has been demonstrated to induce ferroptosis in various diseases. For example, studies show that LPS upregulates ACSL4 expression in esophageal tissue by activating the transcription factor specificity Protein 1 (Sp1), thereby mediating ferroptosis and mucosal injury (Liu et al., 2022b). Similarly, other research confirms that LPS contributes to Pb-induced fatty liver lesions by inducing hepatocyte ferroptosis (Miao et al., 2023). Crucially, LPS has been directly employed to induce ferroptosis in OA models (Zhang et al., 2024b; Zhang X. et al., 2025), supporting its role in this disease. Therefore, LPS likely serves as a pathway linking the gut-joint axis and ferroptosis in OA. Further studies should seek to define the detailed molecular mechanism by which LPS induces ferroptosis in OA (including the pathways that control important molecules such as ACSL4), measure the potential of a pathway like ferroptosis to be targeted therapeutically (for example, by ferroptosis inhibitors or controlling the function of gut barrier) and study its clinical applicability, in particular looking for the correlation between LPS levels and ferroptosis biomarkers (e.g., 4-hydroxynonenal, glutathione) and exposure to OA disease activity in clinical cohorts. Additionally, studies should analyze the synergistic or antagonistic effects of LPS with other microbial metabolites (e.g., SCFAs, TMAO) in the OA microenvironment on ferroptosis regulation. Collectively, these efforts will provide both theoretical foundations for novel OA therapies targeting the gut-joint axis and ferroptosis and evidence for translational medicine to establish a precision treatment system rooted in the “gut-joint axis-ferroptosis” framework.

##### 4.3.2.2 TMAO drives chondrocyte ferroptosis in OA progression

Trimethylamine (TMA), produced by intestinal microbiota metabolism of linoleic acid, glycerophosphocholine, and choline, is subsequently oxidized in the liver to yield TMAO; evidence suggests TMAO contributes to OA pathogenesis by promoting chondrocyte apoptosis and ECM degradation (Wang Y. et al., 2025). Meanwhile, TMAO can significantly upregulate the expression level of PIEZO1 and enhance the sensitivity of chondrocytes to mechanical loading, and this effect may be closely related to the pathogenesis of OA (Zhuang et al., 2023). The above research results confirm that TMAO plays a role in promoting the progression of OA. Notably, *in vitro* experiments have established ferroptosis as a key mechanism for TMAO-mediated renal tubular epithelial cell death (Ge et al., 2024). Therefore, the mechanism by which TMAO promotes the development of OA may involve promoting ferroptosis of chondrocytes. Future studies need to focus more on validating the inductive effect of TMAO on ferroptosis in the *in vivo* OA models and human chondrocytes. It is also very necessary to identify if TMAO contributes to chondrocytes’ injury through modulating the classical pathways of ferroptosis such as ACSL4/GPX4 or other specific pathways, such as those of iron-related proteins, lipid peroxidation products, and other sensitivities to mechanical stimulation. In addition, single-cell sequencing and similar technologies need to be used to unpack differential consequences of TMAO on individual chondrocyte populations. At the same time, this should be accompanied by systematic assays to quantify the protection conferred by intervention strategies aimed at gut microbiota (e.g., inhibiting TMA-producing bacteria), targeting the TMA-TMAO metabolic axis or direct scavenge of TMAO on chondrocyte ferroptosis and OA progression. Last, large clinical cohorts should be used to disclose associations between serum/synovial fluid TMAO levels, ferroptosis markers in the joints (e.g., MDA), and OA grading by radiological or pathological behavior. These efforts will provide new theoretical basis and promising targets for combating OA prevention and therapy following the gut–joint axis.

##### 4.3.2.3 BAMs may promote ferroptosis in OA pathology

Recent studies also brought about important insights on the regulatory role of bile acid metabolites (BAMs) in ferroptosis. For example, a bile acid metabolite (gut microbiome-derived glycochenodeoxycholic acid) activates the TFR/ACSL4 pathway to induce ferroptosis, promoting progression of environmental toxin-associated metabolic dysfunction-associated fatty liver disease (Liu et al., 2022a). Conversely, the bile acid deoxycholic acid (DCA) aggravates colonic inflammation induced by a high-fat diet; its aggravating effect is caused by induction of ferroptosis through the upregulation of expression of HIF-2α and DMT1 (Wang C. et al., 2024). Notably, bile acid signaling pathways can also contribute to OA pathology, as indicated by the alleviation of murine OA after suppression of farnesoid X receptor-the receptor for the bile acid glycoursodeoxycholic acid (GUDCA), which upregulates intestinal glucagon-like peptide-1 secretion (Yang et al., 2025). Taken together, BAMs and other pathways mediated by them, not only regulate ferroptosis, but are also related to OA pathology. However, systematic studies are needed to clarify whether BAMs participate in OA development via the “gut-joint axis,” that is, via a ferroptosis-modulating mechanism. Therefore, more detailed studies will be needed to establish whether BAMs (e.g., GCDCA, DCA, GUDCA) influence the ferroptosis of joint tissue cells, such as chondrocytes and synoviocytes, along this axis, promoting the initiation and progression of OA. Revealing this mechanism reveals new mechanisms of gut-joint crosstalk of OA and offers a groundbreaking theoretical base for prevention and treatment of OA through bowel-inducing the behavioral change for OA by intervening BAMs signaling or ferroptosis inhibition.

#### 4.3.3 Context-dependent dual role of butyrate in modulating ferroptosis via multiple signaling pathways

The direct chondroprotective effects of butyrate in OA are well understood and mainly mediated by the attenuation of inflammation and matrix break down, both of which inhibit cartilage catabolism (Han et al., 2025). At the cellular level, butyrate possesses antioxidant effects that reduce oxidative stress and prevent ferroptosis; thus, butyrate can decrease the cellular level of H₂O₂, which is a major indicator associated with ferroptosis (Hua et al., 2024). Importantly, butyrate regulates ferroptosis in a cell type-dependent manner. In models of acute liver injury, it attenuates ferroptosis via activation of the AMPK/sequestosome 1/NRF2 pathway (Zhang H. et al., 2025), while in cardiomyocytes, ferroptosis is suppressed through downregulation of lipocalin 2 (LCN2) (Zhang et al., 2025b). Conversely, butyrate may promote ferroptosis in specific contexts. In tumor cells, sodium butyrate (NaB) promotes ferroptosis by enhancing ROS production and depleting GSH via the free fatty acid receptor 2/cyclic adenosine monophosphate/ protein kinase A axis (Wang et al., 2023). Similarly, in periodontal tissues, it induces ferroptosis in fibroblasts by activating the HIF-1α/p38 Mitogen-Activated Protein Kinase pathway and enhancing HIF-1α/Cyclin-Dependent Kinase 9/ Bromodomain-Containing Protein 4 interactions, leading to NCOA4-mediated ferritinophagy (Zhao et al., 2020). Although the exact molecular mechanism of the dual regulation of ferroptosis by butyrate has not been fully elucidated, accumulating experimental evidence gradually suggests that it may be involved in the pathophysiological regulation of OA by regulating the ferroptosis process of chondrocytes. Given the dual role of butyrate in regulating ferroptosis, future research should first clarify the specific molecular mechanisms by which butyrate inhibits ferroptosis in OA chondrocytes—such as through the AMPK/NRF2 pathway or downregulation of factors like LCN2—and its interactions with pathological microenvironments including inflammation and ECM degradation. Next, it should explore the OA-specificity of butyrate’s bidirectional regulation of ferroptosis, namely how factors like cell type, concentration, and microenvironmental redox state influence this functional switching. Then, deciphering the “gut microbiota-butyrate-ferroptosis” crosstalk in the gut-joint axis is vital to further understanding the whole process, such as the effects of gut microbiota metabolic functions on intra-articular butyrate and the transmission pathway therein. Additionally, clinical studies are also required to understand whether there is correlation of serum or synovial fluid butyrate levels with ferroptosis markers (e.g., GPX4, ROS) or disease severity in OA patients, and the chondroprotective efficacy of butyrate supply to beneficial bacteria by probiotics or dietary fibers. Finally, investigating butyrate combination therapy strategies (e.g., with anti-inflammatory drugs or ferroptosis inhibitors) and joint-targeted delivery systems (e.g., nanocarriers) to enhance its local bioavailability will be crucial. Collectively, these studies will provide a clearer theoretical framework and translational basis for the precise application of butyrate in OA.

### 4.4 Potential impacts of OA ferroptosis on intestinal homeostasis

In the collagen-induced arthritis mouse model, erastin promoted joint ferroptosis in a peptidylarginine deiminase 4 (PAD4)-dependent manner and exacerbated gut microbiota disturbance and altered gut-derived metabolites; however, the PAD4 inhibitor GSK484 partially attenuated both joint ferroptosis and these altered gut microbiotas (Zhu et al., 2025). Separately, erastin-induced ferroptosis dramatically impaired dysbiosis in the mouse gut microbiota at the phylum and genus levels (Ma et al., 2023), which illustrated the influence of RA’s ferroptosis on the change in the gut microbiota. Analogously, in the context of OA, it is speculated that local ferroptosis in joints may release metabolites such as pro-inflammatory factors and LPO into the bloodstream, thereby regulating the compositional and structural features of the gut microbiota.

Concurrently, substantial evidence confirms that ROS exacerbate systemic inflammation by compromising intestinal mucosal barrier integrity, consequently enabling microbial transmigration with bioactive derivatives across the intestinal barrier into systemic circulation (Zhu et al., 2025). Notably, ferroptosis is characterized by lipid ROS accumulation (Guo et al., 2022) Therefore, we speculate that ferroptosis of OA chondrocytes may release a large amount of ROS into the blood circulation, increasing the level of serum oxidative stress, thereby disturbing the intestinal homeostasis and further exacerbating the pathological process of OA. It is supported by this hypothesized mechanism by one longitudinal clinical cohort study: serum ROS levels were associated with joint space narrowing and its 5-year progression; serum oxidative stress status was positively associated with KOA severity and disease progression in a community-dwelling study population (Ido et al., 2025). The core direction of future research should focus on directly verifying the causal relationship between local joint ferroptosis and gut microbiota dysbiosis through germ-free OA mouse models, fecal microbiota transplantation experiments, or conditional knockout models of key genes for chondrocyte-specific ferroptosis (such as GPX4 and ACSL4). Concurrently, it is imperative to disentangle the concrete regulation on functional subgroups of the gut microbiota induced by metabolites from articular ferroptosis (for example, ROS and LPO) (such as probiotic genus composition, abundance of populations such as those of *Lactobacillus* and the Bacteroidetes/ Firmicutes ratio) and gut structural mucosal barrier integrity (for example, the expression of tight junction proteins, such as occludin and claudin). Thus, it provides a theoretical basis for this hypothesis and also precise treatment for OA.

### 4.5 The “gut microbiota-ferroptosis axis” hypothesis in OA pathogenesis

**Figure 2:**
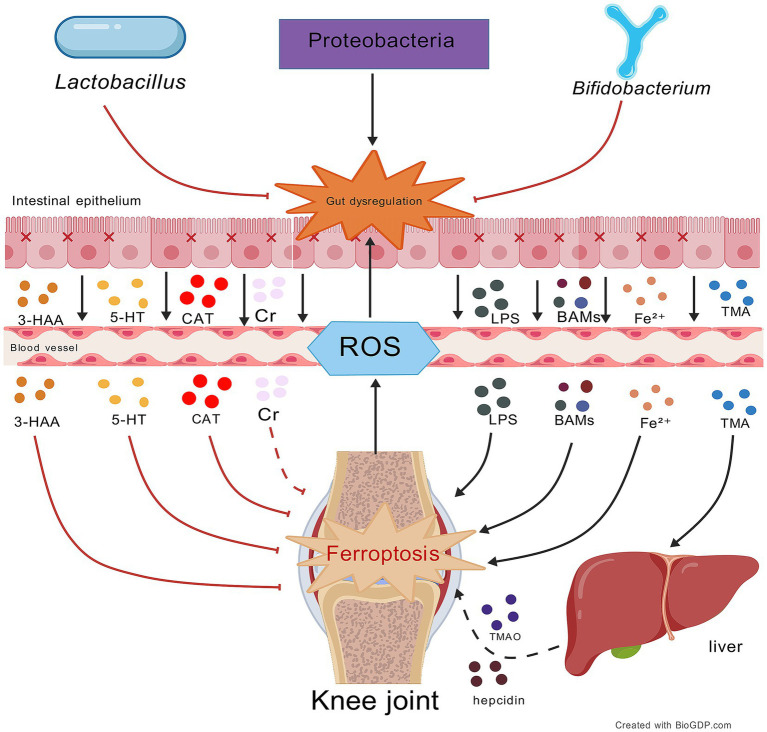
Impact of gut microbiota and their metabolites on ferroptosis in OA. This figure illustrates the bidirectional regulation of OA ferroptosis by gut metabolites via the gut-joint axis: protective metabolites (e.g., Cr, 5-HT, 3-HAA, CAT) inhibit ferroptosis, while harmful metabolites (e.g., GCDCA, LPS, DCA) exacerbate it. Central to this process is gut mi-crobiota balance; beneficial bacteria (e.g., Lactobacillus, Bifidobacterium) support protection and homeostasis, while pathogenic (e.g., Proteobacteria) overgrowth promotes dysbiosis and harmful metabolites. A vicious cycle forms where OA ferroptosis-generated ROS further disrupts the gut microbiota. Black lines: promotion of processes/mechanisms. Red lines: inhibition of processes. Solid lines: experimentally proven relationships. Dashed lines: proposed relationships. Figures all created with BioGDP.com (Jiang et al., 2025).

**Figure 3:**
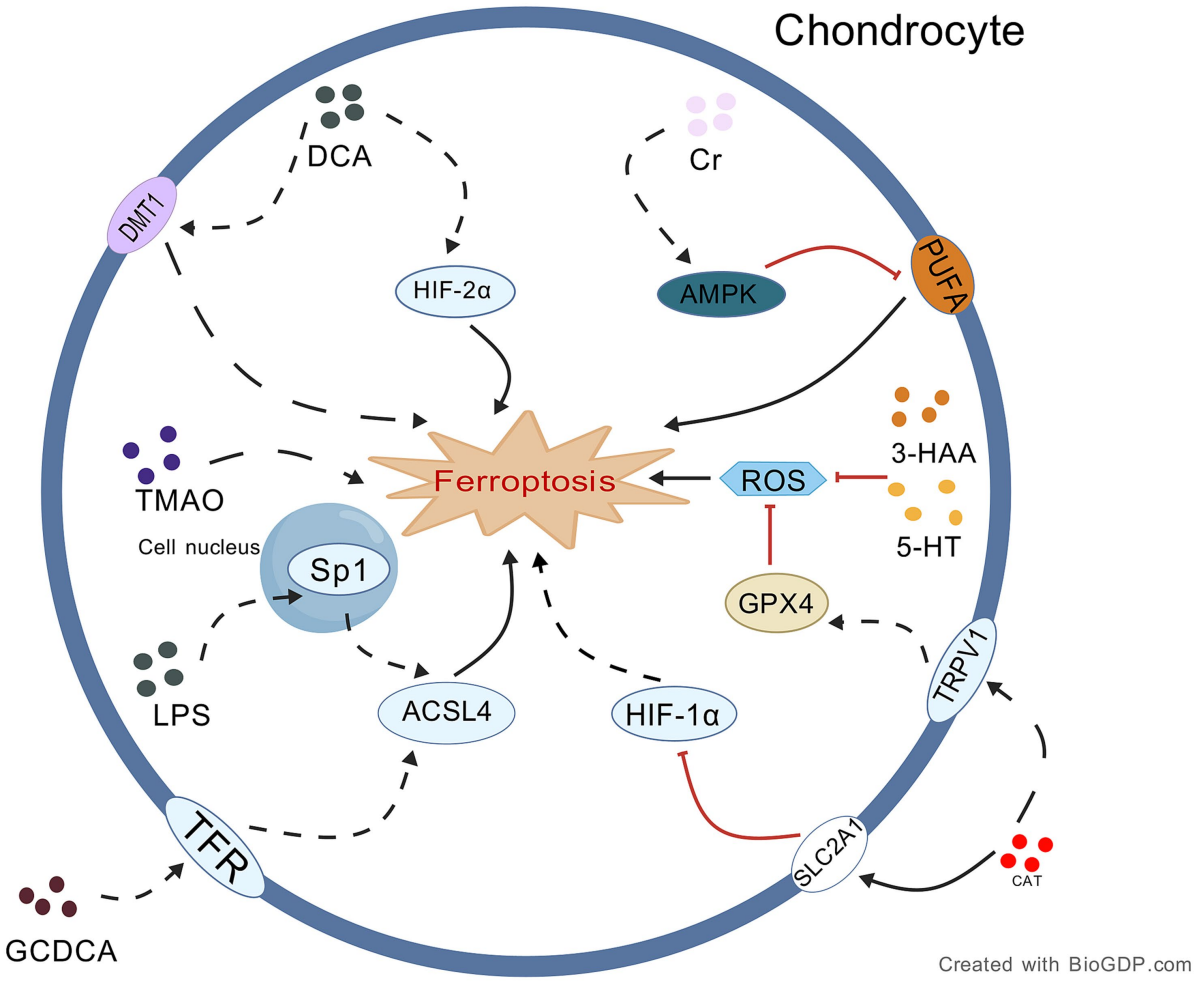
Proposed mechanism underlying the role of intestinal metabolites in OA chondrocyte ferroptosis. This figure delineates the dual-directional regulation of chondrocyte ferroptosis by gut metabo-lites via specific signaling pathways: protective metabolites (e.g., Cr, 5-HT, 3-HAA, CAT) inhibit ferroptosis through mechanisms like AMPK activation and GPX4 upregulation, while harmful metabolites (e.g., GCDCA, LPS, DCA) promote ferroptosis via pathways such as Sp1/ACSL4 and HIF-2α/DMT1 activation. Collectively, these metabolites may form a complex regulatory network targeting key molecules to influence osteoarthritis progression. Black lines: promotion of processes/mechanisms. Red lines: inhibition of processes. Solid lines: experimentally proven relationships. Dashed lines: proposed relationships. Figures all created with BioGDP.com (Jiang et al., 2025).

Elaborating on the above research evidence and theoretical conclusions, we postulate this “gut microbiota-ferroptosis axis” hypothesis to indicate this relationship plays a role in and is a mechanism in the pathogenesis of OA. On one hand, gut dysbiosis facilitates ferroptosis by affecting iron metabolic pathways, like blocking the synthesis and release of hepatic hepcidin and expression of the iron transporter FPN1 to inhibit iron efflux and causing systemic and local iron overload in joints, thus leading to ferroptosis in OA chondrocytes. Meanwhile, iron overload can therefore have a synergistic effect by perturbing the intestinal barrier and exacerbating ferroptosis in OA. On the other hand, it further exacerbates ferroptosis through changes to the metabolite profiles—specifically, reducing the production of anti-ferroptosis metabolites such as 5-HT and 3-HAA, and increasing the abundance of pro-ferroptosis metabolites such as LPS and TMAO. And modifications to the relative abundance of different genera of bacteria intensify such effects: when the relative abundance of protective bacteria (eg, *Bifidobacterium, Lactobacillus sakei*) decreases, their indirect protective effects on ferroptosis lessen, and hence OA is further promoted. Conversely, ferroptosis can feed back into the gut via metabolic products such as ROS through blood to aggravate gut dysbiosis, and then form a self-enhancing “ferroptosis-gut dysfunction cycle” in OA. Combining the different arms of gut microecology, iron metabolism, cell death in this hypothesis, it offers a new explanatory model for investigating “gut-joint axis” in OA and proposes possible targets for OA intervention strategies including changes of gut microbiota or ferroptosis. Future work will need to focus first on testing the mechanistic core elements of the “gut microbiota-ferroptosis axis” hypothesis, interpret the cycle of the “gut microbiota-ferroptosis axis,” and attend to several issues in its clinical translation. First, using germ-free animals, directional microbiota transplantation, and gene editing techniques to clarify the causal mechanisms by which protective genera such as *Bifidobacterium* regulate the “hepcidin-FPN1 axis” and key molecules of chondrocyte ferroptosis (GPX4, SLC7A11), thereby consolidating the logical chain of “microbiota-iron metabolism-cell death.” Second, leveraging longitudinal cohorts and multi-omics technologies to reveal the time-dependent evolution of the “gut dysbiosis-iron overload-ferroptosis-intestinal dysfunction” cycle and identify key windows for early intervention. Third, exploring synergistic intervention strategies targeting gut microbiota (e.g., probiotic combinations) and ferroptosis (e.g., GPX4 activators) to evaluate their synergistic effects on OA cartilage protection. Fourth, verifying the correlation between combined markers—including the abundance of specific genera (e.g., decreased Bifidobacterium), iron metabolism indicators (e.g., elevated serum hepcidin), and pro-ferroptotic metabolites (e.g., increased TMAO)—and OA clinical phenotypes through large-sample clinical studies, and exploring their diagnostic or prognostic value. These directions are the core support for translating the hypothesis from theory to application and directly determine whether the “gut microbiota-ferroptosis axis” mechanism can become a breakthrough framework and intervention target in the study of the “gut-joint axis” in OA (Figures 2, 3).

## 5 Potential therapeutic strategies for OA targeting the “gut microbiota-ferroptosis” axis

Based on the innovative hypothesis of the “gut microbiota-ferroptosis axis,” dual-target compounds such as resveratrol, and salidroside show potential therapeutic value in improving the progression of OA by synergistically modulating the gut-joint axis and inhibiting the ferroptosis cascade reaction. Existing research evidence indicates that such compounds may exert their effects through multiple mechanisms. On the one hand, they can repair the integrity of the damaged intestinal barrier and promote the enrichment and metabolic activity of protective flora (such as *Akkermansia* and *Lactobacillus*), thereby down-regulating the low-grade chronic inflammation in the whole body and local joints through the “gut-joint axis.” On the other hand, they can directly inhibit the iron-dependent lipid peroxidation cascade reaction in chondrocytes (such as regulating the GPX4, NRF2 pathways and the expression levels of proteins implicated in iron metabolism) to block or delay the ferroptosis of chondrocytes. Future research should prioritize the systematic validation of the synergistic therapeutic effects of the above drugs through this signaling axis in OA animal models. Meanwhile, it is urgent to develop cartilage-targeted delivery systems to enhance the bioavailability of drugs in the joint cavity and their specific binding ability to chondrocytes, while avoiding potential interference with the homeostasis of the gut microbiota. For candidate drugs Jingfang Granules that have not been thoroughly explored in the field of OA, standardized OA mouse models should be used for rigorous pharmacodynamic validation. The focus should be on evaluating their regulatory effects on the gut microbiota structure, SCFAs metabolic profile, and cartilage ferroptosis markers (e.g., MDA, iron accumulation levels). These studies will provide crucial experimental evidence for constructing a combined intervention strategy of “gut microbiota-ferroptosis axis,” and contribute to clarifying the causal relationship and molecular synergistic mechanism between gut microbiota dysbiosis and cartilage ferroptosis. Ultimately, they will open up new theoretical and practical paradigms for the precise treatment of OA (Table 1).

**Table 1:** Therapeutic agents targeting the gut microbiota-ferroptosis axis in OA: mechanisms and evidence.

Agent	Effects on gut microbiota/gut barrier	Mechanisms of ferroptosis inhibition	Associated with the hypothesis
Resveratrol	Increase Bacteroides, *Lactobacillus*, and *Bifidobacterium* spp.; enhance microvilli regeneration and mucosal barrier function against LPS (Yu et al., 2025)	Activate sirtuin 3/forkhead box protein O3a axis; upregulate superoxide dismutase 2/catalase; reduce ROS/LPO and enhance GSH/GPX4 activity in OA (Song C. et al., 2025)	Increase the protective flora (*Lactobacillus* and *Bifidobacterium*), enhance the intestinal barrier function, and directly inhibit ferroptosis of OA cartilage
Quercetin	Correct gut dysbiosis and fecal metabolite abnormalities (Ning et al., 2024)	Activate AMPK/NRF2/GPX4 pathway in OA (Dong et al., 2025)	Regulate intestinal dysregulation and metabolites, and directly inhibit ferroptosis in OA cartilage
Ferrostatin-1	Restores microbiota homeostasis and mitigates intestinal barrier disruption post-radiation (Mao et al., 2024)	Inhibits OA chondrocyte ferroptosis by suppressing malondialdehyde/ Fe^2+^ accumulation, lipid ROS, and ECM degradation (Guo et al., 2022)	Restore intestinal homeostasis and barrier, and specifically inhibit ferroptosis in OA through multiple mechanisms
Hesperetin	Enhances microbiota diversity and homeostasis, alleviating experimental colitis (Wang J. et al., 2024)	May inhibit ferroptosis in OA by AMPK-mediated NF-κB suppression (Wu et al., 2021)	Enhance the homeostasis of gut microbiota, restore the diversity of microbiota, and inhibit the AMPK/NF-κB pathway to alleviate ferroptosis in osteoarthritis
Salidroside	Remodel gut microbiota in diabetic mice; reduce systemic iron burden to alleviate cardiomyocyte ferroptosis (Zhang J. et al., 2024)	Activate sirtuin 1/forkhead box protein O1 axis to antagonize LPS-induced ferroptosis in OA (Zhang X. et al., 2025)	Reshaping the gut microbiota reduces systemic iron burden and activates related pathways to inhibit ferroptosis in osteoarthritis
Melatonin	Alleviates intestinal inflammation/barrier damage and reduces LPS synthesis/transport (Miao et al., 2023)	Regulation of NADPH oxidase 4-induced ferroptosis in OA (Wang Q. et al., 2024)	Improve intestinal damage, reduce the entry of harmful metabolites (e.g., LPS) into the blood circulation, and directly inhibit ferroptosis in OA
Jingfang Granules	Restore gut microbiota homeostasis, repair intestinal barrier, and enhance SCFAs production (Wang X. et al., 2025)	Activate AMPK for lipid metabolism, block NOD-like receptor family, pyrin domain containing 3/toll-like receptor 4 /NF-κB to inhibit synovial ferroptosis in RA (Wang X. et al., 2025)	Reshaping gut microbiota homeostasis, Promote the production of protective metabolites (such as SCFAs), inhibit ferroptosis in RA, but no relevant research in OA currently

## 6 Conclusions and outlook

KOA, the most prevalent and representative form of OA, is a globally prevalent and disabling condition; however, the current drugs are limited by the transient procedure and lack of useful methods for delaying disease progress. Recent evidence suggested that iron accumulated LPO triggered ferroptosis as a evolutionally conserved, regulated cell death pathway and mediated chondrocyte loss in OA. Meanwhile, gut microbiota dysbiosis also had strong influence on OA development through the gut-joint axis: intestinal barrier disruption, increased endotoxemia throughout the body, and decreased SCFA all drove joint degeneration. Currently, there is preliminary evidence revealing potential crosstalk between the intestinal microbiota and ferroptosis in OA.

Based on a comprehensive analysis of existing evidence, we postulate the “gut microbiota-ferroptosis axis” hypothesis may play a role in the pathological progression of OA. Specifically, the dysbiosis of gut microbiota and abnormal iron metabolism can promote each other, synergistically facilitating ferroptosis in OA. Meanwhile, when gut microbiota dysbiosis occurs, it may reduce ferroptosis-protective metabolites (such as 5-HT and 3-HAA) and increase ferroptosis-promoting metabolites (such as TMAO and LPS). Through the gut - joint axis, this can decrease the resistance of OA chondrocytes to ferroptosis. Conversely, joint ferroptosis may release harmful metabolites such as ROS into the bloodstream, disrupting gut homeostasis. Thus, a potential self-amplifying cycle may form between ferroptosis and gut dysfunction in OA. To verify this hypothesis, it is urgent to conduct experiments on intestinal microbiota transplantation and tracking of intra - articular ferroptosis markers in OA animal models. Meanwhile, a longitudinal cohort study should be carried out to correlate the dynamics of the patients’ gut microbiota, the levels of serum gut metabolites with the progression of joint ferroptosis.

In summary, clarifying the link between the gut-joint axis and ferroptosis in OA would be of great significance not only for better understanding the pathological processes at multi-levels (local joint tissues, the gut environment, and circulation), but also from a unique angle for guiding novel therapies development in OA Deep investigative approach to understand how major gut bacterial metabolites (tryptophan-derived metabolites, TMAO and so on) modulate ferroptosis in OA is very promising to elucidate the central regulatory mechanism acted by the gut-joint axis on ferroptosis in OA. First of all, we should clarify the detailed molecular mechanisms implicated in ferroptosis and gut-joint axis crosstalk, and investigate their synergy with several candidate therapeutic drugs, such as resveratrol, quercetin, etc. Such knowledge will help us achieve a deep understanding of OA’s complicated pathological processes, provide new insight for developing novel therapeutics targeting this debilitating disease, improve clinical outcome, and patient well-being. Notably, the impact of gut microbiota on joints may be site-specific. This review does not clarify the differences in the effects of the gut microbiota—ferroptosis axis on weight-bearing and non-weight-bearing joints. Load-bearing joints (e.g., knees and hips) are subjected to long-term mechanical stress, and their microenvironment may amplify the effects of oxidative stress and iron deposition triggered by dysbiosis. In contrast, although hand OA is also affected by systemic inflammation, it remains to be explored whether its pathological process is more dependent on specific metabolites (such as TMAO). Therefore, future research needs to compare the activation levels and dominant pathways of the “gut microbiota-ferroptosis axis” in different OA subtypes.
